# Optimization and comparative characterization of neuraminidase activities from *Pseudomonas aeruginosa* with *Klebsiella pneumoniae*, Hep-2 cell, sheep kidney and rat liver lysosome

**Published:** 2010-03

**Authors:** C Ghazaei, M Ahmadi, N Hosseini Jazani

**Affiliations:** 1 Department of Microbiology, Faculty of Veterinary Medicine, University of Urmia, Urmia, Iran; 2 Department of Microbiology, Immunology and Genetics, Faculty of Medicine, Urmia University of Medical Sciences, Urmia, Iran

**Keywords:** *P. aeruginosa PAO1*, *K. pnumoniae*, Neuraminidase, fluorometric assay, specific activity, Lysosome, Iran

## Abstract

**Background and Objectives:**

The properties of neuraminidase produced by *P. aeruginosa* strain PAO1 during growth in a defined medium (BHI) was examined and compared with some neuraminidase features of *K. pneumoniae* in this investigation.

**Materials and Methods:**

The enzyme was isolated from concentrated culture supernatants of *P. aeruginosa* which was used in a sensitive fluorometric assay by using 2′-(4-methylumbelliferyl) α-D-N acetylneuraminic acid as substrate.

**Results:**

Neuraminidase production in *P. aeruginosa PAO1* paralleled bacterial growth in defined medium (BHI) and was maximal in the late logarithmic phase of growth but decreased during the stationary phase, probably owing to protease production or thermal instability. Highest production of *P. aeruginosa PAO1* neuraminidase was in BHI culture media. The neuraminidase of *P. aeruginosa PAO1* possessed an optimum temperature of activity at 56°C and the activity was maximal at pH 5. Heating the enzyme to 56°C for 45 min., in the presence of bovine serum albumin destroyed 33.1% of it's activity and addition of Ca^+2^, EDTA and NANA also decreased activity markedly.

**Conclusion:**

The results revealed that the highest specific activity is for *p. aeruginosa PAO1*.

## INTRODUCTION

*Pseudomonas aeruginosa* is a motile, gram negative, non-spore forming rod shaped bacterium (1, 2) and one of the most important opportunistic pathogens in human and animals. *P. aeruginosa* produces several pathogenic factors that play important roles in the virulence of this microorganism (3, 4). One of these virulence factors is neuraminidase, an enzyme responsible for the cleavage of N-acetylneuraminic acid (NANA) from mucin, glycoproteins, and gangliosides. *P. aeuroginosa* was first noted to produce neuraminidase by Shilo (5, 6).

Intact epithelial cells are relatively resistant to *Pseudomonas* colonization and attachment (5). In vitro studies showed that (7) many pulmonary and gastrointestinal pathogens including *P. aeruginosa* bind to the N-acetylgalactosamine-beta-1-4-galactose (GalNAcβ1,4Gal) moiety exposed on asialylated glycolipids (6, 8), suggesting that the ability to desialylate mucosal surfaces could contribute in bacterial colonization. The GalNAcβ1-4Gal sequence present in asialylated gangliosides can act as a receptor for several pathogens of the respiratory or gastrointestinal tract including *P. aeuroginosa* (5, 6, 8).

Neuraminidase has been implicated as a virulence factor and may serve as a marker for determining virulence of *P. aeuroginosa* strains*.* This enzyme has a key role in the initial stages of pulmonary (7), urinary and gastrointestinal tract infections by targeting bacterial glyco-conjugates and contributing to the formation of biofilm (5, 6).

The aim of the present study was to investigate the details of the production and properties of neuraminidase produced by *P. aeuroginosa* PAO1. Moreover, we wanted to develop an assay and determine best culture conditions with different media including brain heart infusion broth (BHI), Brucella broth (BB), Minimal Media (M9), Pepton water (PW) and Tryptose soy broth (TSB) for neuraminidase production and activity. Meanwhile, the effects of different environmental conditions such as pH, temperature, added cation (Ca^+2^) and components (N-acetyl neuraminic acid, Ethylenediaminetetraacetic acid) on the expression and activity of the neuraminidase were investigated.

## MATERIALS AND METHODS

**Bacterial strains and culture media.** *Pseudomonas aeruginosa* strain PAO1 was kindly provided by Dr. E.A. Worobec (Department of Biology, Faculty of Sciences, University of Manitoba, CA.) and *Klebsiella pneumonia* ATCC 10031 was obtained from the American Type Culture Collection. Bacteria were cultured in brain heart infusion broth (Merk Laboratories, Detroit, Mich) at 37°C with continuous shaking up to the stationary phase of growth, which was monitored by measuring cell turbidity at 600 nm (5, 6, 9). The culture was harvested by centrifugation at 12,000×g for 15 min at 4°C. The supernatant was filtered through a membrane filter (0.45um; Millipore Corp.) and dialyzed against 0.1 M acetate buffer (pH 5.5) for a period of 48 h at 4°C (5, 9, 10).

**Assays for neuraminidase activity**. Neuraminidase activity was measured by fluorescence spectroscopy. This method is the most sensitive and specific method for assay of neuraminidase activity which uses 4-methylumbelliferyl-α-D-N- acetylneuraminic acid (MUN) as substrate (Sigma, St. Louis, Mo.) (4, 11, 12). Upon hydrolysis of MUN by neuraminidase, free N-acetylneuraminic acid (NANA) and 4-methylumbelliferone (4-MU) (Sigma, St. Louis, Mo.) are formed with a shift in the fluorescence spectra (excitation maximum/fluorescence maximum) from 315/374 nm (substrate) to 365/450 nm (product). Enzyme activity is then measured by fluorescence of 4-methylumbelliferone (4-MU) at 450 nm.

The quantitative determination of neuraminidase activity with 2′-(4-methylumbelliferyl)-α-D-N- acetylneuraminic acid (MUN) was detected with an F-2500 fluorescence spectrophotometer Hitachi (fluorimeter) using excitation light at 365 nm and measuring emission light at 450 nm (4, 6, 11, 13). A 10 mM stock solution of 4-methylumbelliferone (4-MU) was diluted to a 0.5 uM standard solution. Serial dilutions were made with 0.1 M sodium acetate buffer (pH 5.5) as a diluent and these data were used to make the standard curve (4). A stock solution of 2′-(4-methylumbelliferyl)-aL-D-N-acetylneuraminic acid (MUN) was prepared in distilled water at a concentration of 110 umol/ml and stored at -20°C in 100 aliquots (4, 13). To quantitatively assay the samples for sialidase activity, 2′-(4-methylumbelliferyl)-a-D- N-acetylneuraminic acid (110 umol/ml) was mixed 1:1 with sodium acetate buffer(0.1 M). Test sample (50 ul) was added to the reaction mixture and incubated at 37°C for 20 min, then 200 ul of a 1.33 M glycine buffer (pH 10.7) was added to the mixture to stop the reaction. A duplicate sample lacking substrate served as negative control (10, 11, 13). Protein concentration was determined using Lowry method with bovine serum albumin (Sigma Total Protein Kit) as standard (4, 10, 13). Specific activity was defined as micromoles of 4-methylumbelliferone (4-MU) formed per milligram of protein per minute at 37°C.

All assays were performed in duplicate. (10, 13) The effect of pH, temperature, added cation (Ca^+2^) and compounds such as N- acetylneuraminic acid (NANA) and ethylenediaminetetraacetic acid (EDTA) on neuraminidase activity, as well as the effect of pH, Ca^+2^ and ethylenediaminetetraacetic acid (EDTA) on extracellular and cell-bound neuraminidase activity was tested in the system described above. Also, the relationship between *P. aeruginosa* PAO1 growth curve and neuraminidase production, the effect of pH and temperature on the stability of enzyme and influence of growth medium on the production of neuraminidase by *P. aeruginosa PAO1* was examined.

**Relationship between *P. aeruginosa PAO1* growth curve and neuraminidase production.** *P. aeruginosa* PAO1 was grown in BHI broth, at 37°C. At given intervals (1 hour), a portion of culture supernatant was removed, and bacterial growth was measured at 600 nm by a biophotometer (Eppendorf BioPhotometer plus). A portion (50 µl) of the cultures supernatant was filtered through a membrane filter (0.45um; Millipore Corp) and dialyzed against acetate buffer (0.1 M) and the activity of enzyme was determined (14–16).

**Activity of secreted and cell bound neuraminidase.** To determine the amount of secreted and cell-bound neuraminidase of *P. aeuroginosa* PAO1 and *K. pneumoniae* ATCC 10031, the cultures of these organism were harvested by centrifugation at 12,000×g. The cell pellet was suspended in phosphate-buffered saline (PBS; 0.01 M [pH 7.4]) to 1:25 of the original volume. The suspension was divided into three parts and treated as follows: (i) Tween 80 (0.1%) added to the cell suspension and the mixture was shaken at 30°C for 30 min, (ii) the cells in phosphate-buffered saline (PBS) suspension were lysed by twice freezing and thawing the suspensions in liquid nitrogen, which were allowed to thaw completely before re-freezing (17) and (iii) the cell suspension in phosphate-buffered saline (PBS) was shaken at 30°C for 30 min. These suspensions were further centrifuged at 27,000*g* for 8 min at 4°C. The supernatant from each portion was passed through a millipore filter (pore diameter, 0.45, um), dialyzed against acetate buffer (0.1 M) and assayed for neuraminidase activity (9, 14, 17).

**Lysosomal neuraminidase assay.** The crude lysosomal fraction (CLF) is obtained after removal of nuclei, cell debris and fat layer by serial centrifugations (18). The enzyme activity of the subcellular lysosomal fraction was measured after disruption of lysosomal particles by freezing and thawing ten times (18–20).

**Hep-2 cell neuraminidase activity.** The cells were trypsinized and growth medium was added with 10% fetal calf serum, and centrifuged for 5 minutes at 600×g. The cells were resuspended in ice cold phosphate buffered saline (PBS) and centrifuged for 5 minutes at 600×g at 4°C and repeating the wash step once again. Supernatant was discarded in all of these steps. The packed cell was filled with extraction buffer and vortexed to achieve an even suspension**.** The cells were broken in a 7 ml Dounce homogenizer using Pestle B (small clearance**).** After every 5 strokes with the pestle, the cells were checked under a microscope using Trypan Blue staining to ascertain the degree of breakage. The supernatant from this portion was passed through a Millipore filter (pore diameter, 0.45 um) and assayed for neuraminidase activity (17, 20).

## REULTS

**Effect of various concentrations of NANA on enzyme production by *P. aeruginosa* PAO1 in minimal media (M9).** Data showed that a 4 mg concentration of N-acetyl neuraminic acid (NANA) has the greatest effect on production of neuraminidase by *P. aeruginosa* PAO1. Further incremental increases had no effect on enzyme production.

**Fig. 1:**
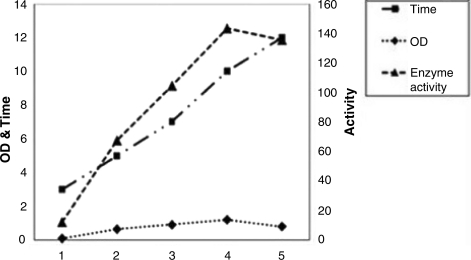
Relationship between extracellular neuraminidase production and bacterial growth for *P. aeruginosa* PAO1.

**Influence of growth medium on production of neuraminidase.** The greatest enzyme yield was in BHI containing complicated components whereas less enzyme was produced in minimal media (M9) media. The pattern observed for neuraminidase production closely paralleled the growth curve of the organism (Fig. 1). The results indicate that the enzyme production is maximal at 37°C in contrast to the lower yield at 42°C & 22°C.

**The relation between enzyme concentration and neuraminidase activity of *P. aeruginosa* PAO1.** The pattern observed for neuraminidase activity closely paralleled the total amount of enzyme. The enzyme activity increased up to a concentration of 0.35 mg/ml protein and followed an almost constant linear rate.

**Relationship between incubation time & neuraminidase activity of *P. aeruginosa* PAO1.** As the incubation time increased, the activity of the enzyme also increased and a linear pattern was maintained for 20 mins. Under the conditions of the assay, 4-methylumbelliferone(4-MU) release decreased within 40 to 60 min. The assay is linear with respect to enzyme concentration.

**The optimum pH and temperature of *P. aeruginosa* PAO1 neuraminidase activity.** The optimum temperature and pH was determined using 4-methylumbelliferyl-α-DN- acetylneuraminic acid (MUN) as the substrate. A peak of maximal activity occurred at 56°C, but greater than 95% of the enzyme activity was destroyed at 65°C. Optimal activity was achieved at pH 6.4.

**Thermal stability of *P. aeruginosa* PAO1 neuraminidase.** Heating the enzyme in the presence and absence of albumin led to approximately 30% and 42.5% reduction of the *P. aeruginosa* PAO1 neuraminidase activity respectively within 45 min at 56 °C.

**Stability of *P. aeruginosa* PAO1 neuraminidase at different pH after 1 hour.** Incubation of the enzyme at various pH showed that the lowest and highest stability of the enzyme occurred at pH 7 and 2 respectively.

**Effects of Ca ^2+^ ions, ethylenediaminetetraacetic acid (EDTA) and N-acetyl neuraminic acid (NANA) on neuraminidase activity of *P. aeruginosa PAO1*.** Added cations and other reagents were examined for their effects on the neuraminidase activity. Calcium chloride (Ca^2+^), ethylenediaminetetraacetic acid (EDTA) and N-acetyl neuraminic acid (NANA) led to a decrease in activity. The results indicated that calcium ions, ethylenediaminetetraacetic acid (EDTA) and N-acetyl neuraminic acid (NANA) have inhibitory effects on the enzyme activity (Table 1).

**Table 1:** Effect of Ca^2+^, EDTA and NANA on neuraminidase activity of *P. aeruginosa* PAO1*.*

Additive	Percent decrease of enzyme Activity
2 mM CaCl_2_	36.2
20 mM CaCl	58.31
0.05 mM EDTA	49.78
0.5 mM EDTA	36.66
10 mM NANA	39.8
20 mM NANA	54.8
40 mM NANA	63

**Presence of intracellular, extracellular and cell-bound neuraminidase of *P. aeruginosa PAO1*.** The supernatant fraction possessed a specific activity of 4.06 µmol of 4-methylumbelliferone released per min per mg of protein. In contrast the whole-cell fraction included cell-bound neuraminidase, treated with shaking and tween to release the intracellular neuraminidase possessed a lower specific activity (Table 2).

**Table 2:** Neuraminidase activity (secreted, intracellular and cell- bound) in *P. aeruginosa* PAO1

Fraction	*Specific activity
Extracellular	4.06
Intracellular	2.32
Cell-bound (treated with shaker)	0.58
Cell-bound (treated with tween)	0.79

* Specific activity is expressed as micromoles of 4-methylumbelliferone released per minute per milligram of protein.

**Fig. 2:**
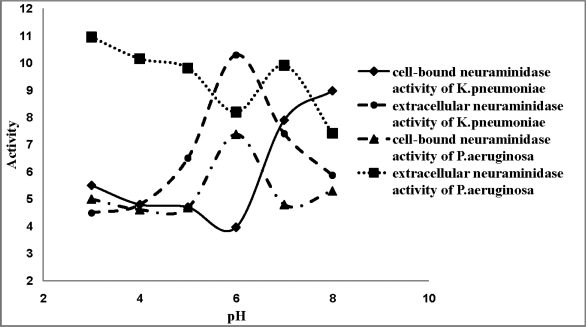
Effect of pH on extracellular and cell-bound neuraminidase activity of *P. aeruginosa PAO1 and K. pneumoniae* 10031.

**Fig. 3:**
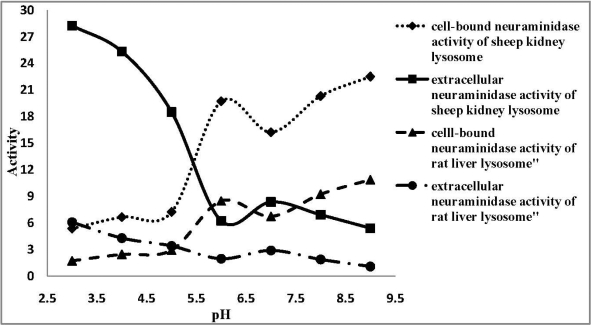
Effect of pH on extracellular and cell-bound neuraminidase activity in sheep kidney and rat liver lysosome.

**Effect of pH, ethylenediaminetetraacetic acid (EDTA) and Calcium chloride (CaCL2) on extracellular and cell-bound neuraminidase activity.** Figs. 2 – 3 show the effect of pH on extracellular and cell- bound neuraminidase activity in *P aeruginosa* PAO1 and *K. pneumonia* ATCC 10031. In *P. aeruginosa* PAO1, increasing the pH led to an initial drop in activity at pH 6 followed by an increase whereas in *K. pneumoniae* ATCC 10031, the enzyme activity increased with increasing pH and an appreciable drop in activity was observed at pH 7. As well, there were some observed increases and decreases in enzyme activity when the whole-cell bacteria was incubated in the presence of various CaCl_2_ and EDTA concentrations for the cell-bound and extracellular neuraminidase activity of sheep kidney and rat liver lysosome (Figs. 2 – 3).

**Neuraminidase specific activity for *P. aeruginosa* PA01, *K. pnumoniae* ATCC 10031, Hep-2 cell, sheep kidney and rat liver lysosome.** The greatest neuraminidase specific activity was found in *P. aeruginosa* PAO1, whereas the lowest was for Hep-2 cell.

## DISCUSSION

Unlike M-9 medium, neuramindase was produced when *P. aeruginosa* PAO1was grown Brain Heart Infusion Broth, Brucella Broth or Pepton Broth without addition of N- acetyl neuraminic acid to the these media. These media contain complex materials such as glycoproteins and other carbohydrate component that are capable to induce enzyme production. Similarly, the enzyme which plays a key role in pathogenesis of bacteria is always produced in different body tissues since they consist of a large amount of sialic acid components (21, 22).

The pattern observed for production of the enzyme in defined medium at 37°C paralleled the growth curve of the organism. For *Streptococcus pneumonia* (10), there is a similar pattern, whereas in *Vibrio cholerae*, production of the enzyme in the stationary phase is more than the other growth phases of bacteria (9, 14).

The destruction of neuraminidase activity during the stationary phase of growth in *P. aeruginasa* PAO1 is probably due to thermal instability of the enzyme or owing to proteolytic enzyme (protease) production which has occurred at the stationary phase and causes breakup of the enzyme (10).

It was indicated that the production of the enzyme for *P. aeruginosa* PAO1 in 37°C is more than the other temperatures. In the *Streptococcus pneumonia*, production of the enzyme is equal at 22 and 37°C (23). The highest rate of enzyme activity was established with 0.25 mg/ml of protein within 20 min and then it is remained at a almost constant linear rate due to this reason. The total amount of the enzyme is too low to permit acting as a substrate. On the other hand, the data suggest that the incubation for various periods of time at 37 °C (10, 20, 30, 40, 50 & 60 min), a gradual decrease in activity at times of 30,40,50 and 60 min which could be due to thermal instability upon prolonged periods of time (14).

The highest activity of the enzyme occurred at 56°C, with 100% of the enzymatic activity destroyed within 20 min at temperature of 65°C which is due to thermal instability of the enzyme. Also, the optimal temperature of activity for *Streptomyces viridifecians* is 56°C (24). Neuraminidase of the group A streptococci is completely inactive after 10 minutes at 50°C (15). Our data shows that some of proteins such as bovine serum albumin are capable to protect the enzyme against temperature denaturation effects (25), so that if they are added into the medium, the enzyme loses just 33.1% of it's activity after 45 min. Neuraminidase of the *Corynebacterium diphtheriae* with serum albumin is completely inactive at temperature of 56°C within 1 h (25).

The enzyme possessed a pH optimum of 5. This pH is included within the optimum pH spectrum of the bacteria neuraminidase (4.5 to 5.6). Beside pH effect on activity of the enzyme, there may be changes in enzyme structure that affects the enzyme stability.

Based on these data, it was revealed that neuraminidase of *P. aeruginosa* PAO1 is susceptible to Ca^2+^. The Calcium ions have increasing effects on the activity of neuraminidase of *Vibrio cholerae* and are capable to make activity of the enzyme two-fold at a concentration of 1mM (24). Adding calcium ions had no effect on neuraminidase activity of *Bacteroides fragilis* (26), *Streptomyces albus* (27), *Corynebacterium diphtheriae* (3), in contrast the neuraminidases from *Mannheimia haemolytica*, *Clostridium perfringens*, and the group A *Streptococcus* also has showed enhanced activity upon the addition of Ca^+2^ (3). The neutral salts such as calcium chloride have direct effects on the enzyme conformation that affects catalytic activity of the enzyme. The reason for enzyme conformational altering by salts is due to the fact that they react with the bipolar bounds existance in the polypeptide chain.

The neuraminidase of *P. aeruginosa* PAO1 was susceptible to EDTA. Neuraminidase of *Vibro cholerae* is susceptible to EDTA and decreases its activity (24) but the neuraminidase activity of *Mannheimia haemolytica* was increased by the addition of EDTA (3). EDTA has not effect on neuraminidase activity of the *Streptomyces* (27). Therefore the neuraminidase of *P. aeruginosa* PAO1 shows the particular pattern regarding to the reaction to calcium ions & EDTA. The preventing effects of N-acetylneuraminic acid is due to structural analogy with enzyme substrate (sialic acid) that competitively prevents activity of the enzyme (13).

The effect of pH on cell-bound or extracellular form of the enzyme is probably due to changing in the environmental electric charge (28, 29). With increasing of pH, the amount of the positive existing charges decreases in the environment and is added to the negative charges (28). If the electrostatic force is the only reason for binding of the enzyme, with increasing of pH, the amount of secreted enzyme should be increased as it is observed at the hexsokinase of mitochondria, while it is not in such manner (25). Such process could be due to heterogeneity and complication of the membrane system in these organisms, and pH is not only effective factor for binding onto or releasing from the membrane (25). On the other hand, the stability of these two enzymes could be different in several pH.

The lower concentration of calcium chloride (less than 0.03M) was induced to increase of enzyme reaction to membrane and being bound to it. But at the concentration more than 0.03 M, the salt had destructive effects over this reaction and was induced to release the enzyme from the membrane. Divalent cations such as Mg^+2^ at the concentration less than 0.02 M cause the increase binding the hexokinase into the mitochondria membrane (25). These two apposite effect could be explained in this way that at the low concentration of salt, the divalent cations are responsible to cover the negative charges over the enzyme or membrane so that they decrease the electric repellent force between the enzyme & membrane causing to make more affinity of enzyme into the membrane (9, 25).

Soluble form of the enzyme at the high concentrations of salt, is due to change in the enzyme conformation and at the tertiary structure of it that affects on catalytic activity of the enzyme as well. The amount of conformational change established by salt is depend on their reaction with the polar bounds at the peptide sequence (25, 30).

Also similarly to Ca^2+^, the opposing effects of EDTA (more and less than 1.5 mM) could be explained by EDTA acting as a chelating agent and resulting in a loss of positive ions, causing an increase in the ionic repellent forces between the enzyme and membrane and consequently becoming soluble. At concentration of more than 1 mM, the enzyme adhesion is due to the increasing ionic forces of solution (9, 25).

Taken together, these results suggest four contrasting organisms. The highest activity is for neuraminidase of *P. aeruginosa* PAO1 and it is 1.82, 11.94, 8.63 and 40.6 fold of *Klebsiella pneumonia* ATCC 10031, sheep kidney, rat liver and Hep-2 cell neuraminidase specific activity respectively and the neuraminidase of *P. aeruginosa* PAO1 is more an extracellular enzyme than cell-bound.
